# Clinical effects of Daole analgesia meter combined with Daole accompaniment on labor pain and delivery outcome in high-risk factors parturients

**DOI:** 10.12669/pjms.41.8.11467

**Published:** 2025-08

**Authors:** Huihui Hao, Bing Sun, Xiaoli Zhu, Jing Zhao, Tian Zhang

**Affiliations:** 1 Huihui Hao Department of High-risk Obstetrics, Baoding Maternal and Child Health Hospital, Baoding 071000, Hebei, China; 2 Bing Sun Department of Nursing, Baoding Maternal and Child Health Hospital, Baoding 071000, Hebei, China; 3 Xiaoli Zhu Department of Delivery Room, Baoding Maternal and Child Health Hospital, Baoding 071000, Hebei, China; 4 Jing Zhao Department of Nursing, Baoding Maternal and Child Health Hospital, Baoding 071000, Hebei, China; 5 Tian Zhang Department of Obstetrics and Gynecology, Baoding Maternal and Child Health Hospital, Baoding 071000, Hebei, China

**Keywords:** Analgesic effect, Delivery outcome, Daole analgesia meter, Daole accompaniment, High-risk factors Parturients

## Abstract

**Objective:**

To explore the application effect of the Daole analgesia meter combined with Daole accompaniment in High-risk factors Parturients.

**Methods:**

This was a retrospective study. Sixty High-risk factors Parturients admitted to Baoding Maternal and Child Health Hospital from January 2023 to October 2024 were selected, among which 30 parturients who were voluntarily treated with Daole analgesia meter combined with whole-process Daole accompaniment were assigned to an observation group, and another 30 parturients undergoing traditional natural delivery without analgesia or accompaniment during the same period were assigned to a control group. The duration of each labor stage, labor pain score, the psychological state during labor [Self-rating Anxiety Scale(SAS) score and Childbirth Attitude Questionnaire(CAQ) score], postpartum hemorrhage within two hours after delivery, and postpartum satisfaction were observed in the two groups.

**Results:**

The first-stage, second-stage and overall labor duration of the observation group were all shorter than those of the control group, with statistically significant differences (*P*< 0.05), the third-stage labor duration showed no statistically significant differences between the two groups (*P*> 0.05). The Visual Analog Scale (VAS) scores in the observation group were all significantly lower compared with the control group at the same stage. The observation group exhibited significantly lower SAS and CAQ scores and volume of postpartum hemorrhage within two hours after delivery than the control group, the differences were statistically significant (*P*< 0.05).

**Conclusion:**

A Daole analgesia meter combined with Daole accompaniment can significantly shorten the labor duration of High-risk factors Parturients, improve the analgesic effect, and increase the natural delivery rate.

## INTRODUCTION

Pain has been identified as the fifth major vital sign along with body temperature, respiration, pulse and blood pressure. Labor pain is a complex and multifactorial experience, mainly caused by the propagation of electrical impulses that are generated by nerve endings during paroxysmal muscular contractions of the uterus and fetus delivery through the birth canal along the lumbosacral plexus to the spinal cord and the pain center in the brain.^1^ Pain intensity is considered to be related to factors such as maternal pain threshold and the number of deliveries.^2^ Labor pain of parturients may induce increased oxygen consumption, hyperventilation and respiratory alkalosis, as well as sympathoexcitation and catecholamine release, resulting in reduced uteroplacental perfusion and prolonged labor.^3^

High-risk factors Parturients are prone to risk events during labor due to their special physical functions or psychological states, and their probabilities of adverse pregnancy outcomes are higher in comparison to normal parturients. Effective analgesic methods can alleviate labor pain and are of great significance in enhancing the confidence of High-risk factors Parturients in delivery. Daole labor analgesia is a non-pharmacological analgesic technique and novel non-invasive delivery method that can provide effective psychological and physiological support for parturients.^4^ With Daole accompaniment, labor is accompanied by professional midwives throughout its entire process, which can effectively alleviate the anxiety and tension of parturients and assist them in passing through this process successfully. The present study intended to explore the clinical efficacy of Daole analgesia meter combined with Daole accompaniment on High-risk factors Parturients through implementing different labor pain management in 60 High-risk factors Parturients admitted to Baoding Maternal and Child Health Hospital.

## METHODS

This was a retrospective study. Sixty High-risk factors Parturients admitted to the Department of Obstetrics in Baoding Maternal and Child Health Hospital from January 2023 to October 2024 were selected as subjects. Data were retrieved from the hospital information and management system. Collected their various information of High-risk factors Parturients , included main clinical features. They were divided into two groups based on voluntary choice of delivery mode, among which 30 parturients who were voluntarily treated with Daole analgesia meter combined with whole-process Daole accompaniment were assigned to an observation group, and another 30 parturients undergoing traditional natural delivery without analgesia or accompaniment during the same period were assigned to a control group.

### Ethical approval:

The study was approved by the Institutional Ethics Committee of Baoding Maternal and Child Health Hospital (No.: 2024-01-K0013; Date: October 31, 2024), and written informed consent was obtained from all participants and their families.

### Inclusion criteria:

- Parturients had at least one maternal high-risk factor^5^, such as pregnancy-induced hypertension (PIH), gestational diabetes mellitus (GDM), placenta previa, polyhydramnios, age over 35 years, etc., and entered the active stage.
- Parturients were confirmed by examination to meet the criteria for vaginal delivery.
- Parturients voluntarily participated in the study, were well informed and able to communicate well with researchers and complete this study according to relevant requirements.

### Exclusion criteria:

- Mental and behavioral abnormalities.
- Cognitive impairment.
- Abnormality of the birth canal, polyembryony and an unwillingness or inability to cooperate.

The control group was not given any analgesia and received routine care and health education for natural delivery in our hospital. Parturients entered the labor room for fetal heart rate monitoring after experiencing regular uterine contractions or premature rupture of membranes. After the uterine cervix was fully opened, fetuses were delivered by midwives in the delivery room. Without abnormal conditions such as hemorrhage during two hours postpartum observation, the parturients were sent back to the ward.

The observation group was treated with Daole analgesia meter combined with whole-process one-to-one Daole accompaniment. A GT-4A Daole analgesia meter (Beijing Benran Tiandi Medical Technology Co. Ltd.) was adopted. Parturients entered the active stage of labor when the cervix was opened about 3 cm. At this time, corresponding electrode pads were attached to their hands and waist by professional midwives, with current intensity set at 0.1-0.3 mA, which should be tolerable and could lead to muscle tremors and tingling sensations. It was stopped when the cervix was fully opened. Meanwhile, Daole accompaniment was provided by one professional midwife throughout the entire process of labor.

During the first stage of labor, the midwife actively communicated with parturients to understand their psychological states and popularize knowledge about natural delivery, played light music to help them relax and alleviate their negative emotions, and guided them to supplement energy at uterine contraction intervals. In the second and third stages of labor, vital signs of the parturients such as blood pressure, fetal heart rate and pulse were monitored after admitted to the delivery room. When regular uterine contractions occurred, the midwife guided the parturients to practice Lamaze breathing and massaged their waist appropriately to relieve pain.

After the full opening of the uterine cervix, the midwife guided the parturients to breathe scientifically, assisted them in adjusting the way they exerted abdominal force, informed them of their delivery process, and continuously encouraged them. After delivery, the midwife assisted the parturients in skin-to-skin contact with newborns for > 30 minutes, praised and affirmed the efforts they made, and performed postpartum observation for two hours. After the maternal and infant conditions stabilized, the midwife returned to the ward with the parturients together and taught them breastfeeding techniques.

### Observation indicators:

Comparison of labor duration: The first-, second- and third-stage labor duration and overall labor duration were recorded and compared between the two groups.

### Comparison of labor pain score:

The intensity of labor pain in each stage of labor was measured using the Visual Analog Scale(VAS)^6^, with a total score of 10. The score was negatively correlated with pain intensity: a score of 1-3 was defined as mild pain, 4-6 as moderate pain, and 7-10 as severe pain.

***Comparison of psychological state during labor and postpartum hemorrhage within two hours:*** Psychological states during labor of the two groups were assessed using the Zung Self-rating Anxiety Scale (SAS)^7^ and the Childbirth Attitude Questionnaire (CAQ)^8^. The SAS consisted of 20 questions, and its total score was negatively correlated with parturients’ state. The lower the score, the better the psychological state. The CAQ had a maximum score of 64, with a higher score indicating higher levels of anxiety and fear. The overall volume of postpartum hemorrhage was recorded within two hours after delivery.

### Comparison of adverse delivery outcomes:

The incidences of postpartum urinary retention, neonatal asphyxia and transfer to cesarean section were observed and compared between the two groups.

### Postpartum satisfaction survey:

On the day after delivery, parturients in both groups were followed up using a postpartum satisfaction questionnaire to understand their levels of satisfaction with perinatal nursing quality. The questionnaire included 10 self-designed items, and parturients’ satisfaction with perinatal nursing quality was classified into three levels: very satisfied, satisfied, and dissatisfied. This questionnaire was filled out by parturients independently or assisted by visiting nursing staff after inquiry. Overall satisfaction rate = (the number of very satisfied cases + the number of satisfied cases) / the number of total cases × 100%. Postpartum satisfaction was compared between the two groups.

### Statistical Analysis:

The data were statistically analyzed using SPSS 24.0 software. Measurement data were expressed as (`x ± s), and enumeration data were described by rate (%). The t test and *χ*² test were utilized for comparisons. P < 0.05 was considered as statistically significant.

## RESULTS

Comparison of general data including age, body mass index (BMI) and gestational age between the two groups revealed no statistically significant differences (*P* > 0.05), as seen in Table-I. The comparison results of the duration of each labor stage between the two groups are shown in Table-II. The comparison results of the intensity of labor pain between the two groups are displayed in Table-III.

**Table-I:** Comparison of general data between the two groups.

Group	Age (*χ̅*±*S*, year)	BMI (*χ̅*±*S*, kg/m^2^)	Gestational age (week)
Control group (n = 30)	30.94±5.01	27.95±4.52	39.12±1.56
Observation group (n = 30)	31.12±5.13	28.06±4.97	38.94±1.49
*χ*^2^/*t*	1.053	0.357	1.432
*P*	0.349	0.708	0.159

**Table-II:** Comparison of labor duration between the two groups (min, *χ̅*±*S*).

Group	First-stage labor duration	Second-stage labor duration	Third-stage labor duration	Overall labor duration
Control group (n = 30)	567.13±64.52	105.80±35.26	16.93±2.87	689.68±70.56
Observation group (n = 30)	505.29±54.89	70.31±34.15	16.46±2.39	592.06±62.35
*t*	4.057	4.508	6.204	6.673
*P*	< 0.001	< 0.001	0.069	< 0.001

**Table-III:** Comparison of labor pain intensity between the two groups (score, *χ̅*±*S*).

Group	First stage of labor	Second stage of labor	Third stage of labor
Control group (n = 30)	8.53±1.42	7.80±1.22	6.57±1.14
Observation group (n = 30)	4.09±1.09	3.51±0.95	2.46±0.31
*t*	17.057	14.532	16.225
*P*	< 0.001	< 0.001	< 0.001

Comparison of psychological state and postpartum hemorrhage Compared with the control group, the observation group exhibited significantly lower SAS and CAQ scores during labor and significantly lower volume of postpartum hemorrhage within two hours after delivery, and the differences were statistically significant (*P* < 0.05) Table-IV. The incidence of adverse delivery outcomes such as postpartum urinary retention, neonatal asphyxia and cesarean section in the two groups were listed in Table-V. The comparison results of postpartum satisfaction between the two groups were presented in Table-VI.

**Table-IV:** Comparison of psychological state and postpartum hemorrhage between the two groups.

Group	SAS score	CAQ score	Postpartum hemorrhage (ml)
Control group (n = 30)	58.16±5.13	42.04±5.74	215.31±25.42
Observation group (n = 30)	49.93±5.05	23.29±5.39	151.85±23.69
*t*	8.357	10.002	17.291
*P*	0.02	< 0.001	< 0.001

**Table-V:** Comparison of adverse delivery outcomes between the two groups [n (%)].

Group	Postpartum urinary retention	Neonatal asphyxia	Cesarean section	Overall incidence
Control group (n = 30)	2(6.67)	2(6.67)	5(16.67)	9(30.00)
Observation group (n = 30)	1(3.33)	0	1(3.33)	2(6.67)
*t*				2.471
*P*				0.003

**Table-VI:** Comparison of postpartum satisfaction between the two groups.

Group	Very satisfied (n)	Satisfied (n)	Dissatisfied (n)	Overall Satisfaction rate [n (%)]
Control group (n = 30)	11(36.67)	10(33.33)	9(30)	21(70.00)
Observation group (n = 30)	20(66.67)	8(26.67)	2(6.66)	28(93.33)
*t*				1.872
*P*				0.02

## DISCUSSION

In this study, no significant differences were found in general data between the two groups (*P* > 0.05). Daole analgesia meter combined with Daole accompaniment could enhance the comfort and adaptability of High-risk factors Parturients, inspire their confidence in delivery, accelerate their labor, significantly decrease their first-stage, second-stage and overall labor duration, significantly improve their tension and anxiety, and reduce their postpartum hemorrhage volume two hours after delivery, which had statistically significant differences from the control group (*P* < 0.05). In the control group, the parturients exhibited higher incidences of severe pain and transfer to cesarean section during labor due to their regular uterine contractions, cervical dilation caused by descending of the fetal head, excessive traction of the pelvic floor peritoneum, significant anxiety and fear, autonomic nerve hyperexcitability, as well as reduced pain threshold. In the observation group, the parturients showed a lower incidence of severe pain, which was attributed to effective pain management and Daole accompaniment, and their VAS scores and incidences of adverse delivery outcomes were significantly lower compared with the control group, with statistically significant differences (*P*<0.05). Thiel et al.^9^ have demonstrated that negative emotions of parturients can affect the intensity of uterine contractions, prolong labor, and increase the incidence of transfer to cesarean section. Therefore, strengthening psychological intervention for parturients is particularly important. Daole accompaniment enables parturients to have a full understanding of natural delivery, and maintain good psychological preparation and emotional control ability through positive psychological education, assistance in pain management and guidance of Lamaze breathing and relaxation techniques, which can alleviate their negative emotions and pain to a certain extent, and effectively improve their muscle control ability and pain tolerance, so that they can effectively exert force during labor to avoid adverse impacts on the labor process due to force of labor and psychological factors.^10^ Its combined application with the Daole analgesia meter has achieved positive results in the labor of primiparas and older parturients^11^, but its application in High-risk factors Parturients has rarely been studied.

Moreover, the postpartum satisfaction of the observation group was significantly higher than that of the control group, and the difference was statistically significant (*P*<0.05). These results indicate that the combination of Daole analgesia meter and Daole accompaniment can accelerate the labor process, effectively improve parturients’ emotions on the basis of effective pain management, and reduce their fear of delivery. With one-to-one Daole accompaniment, the midwife can popularize measures for relieving pain and breathing correctly to parturients, and praise or comfort them to enhance their confidence, reflecting the concept of “human-centered” midwifery and nursing, which plays an important role in ensuring maternal and infant safety and improving delivery outcomes. Labor pain is a type of physical pain unrelated to diseases, which is an important component of maternal labor and the main cause of negative emotions such as tension and anxiety during labor.^12^-^14^ Labor pain during the first stage is mainly visceral pain, which is principally caused by the fact that uterine contractions lead to the release of mediators such as bradykinin, 5-hydroxytryptamine and histamine, dilatation of the internal cervical os stimulates mechanoreceptors and noxious stimuli are transmitted from sensory nerve fibers accompanying the sympathetic nerve endings to the lumbar sympathetic plexus through the pericervical area, pelvic cavity and lower abdomen, finally to the spinal cord.

In the second stage of labor, dilation and transient ischemia occur in the vagina and vulva as the fetal head descends, and physical pain stimuli are transmitted to the spinal cord through pudendal nerves.^15^ In addition, pain during labor can cause sympathoexcitation, inhibit uterine contractions and delay cervical dilation, thus leading to uterine inertia, decreased confidence in delivery and aggravated anxiety in parturients, which may further lower their pain threshold and increase their pain intensity.^16^ The cesarean section rate in China is as high as 46.2%, and about 45% of High-risk factors Parturients undergo cesarean section due to the fear of pain. Consequently, reducing labor pain becomes a hot research topic in obstetrics. Promoting safe and effective labor analgesia can help reduce the cesarean section rate of High-risk factors Parturients and improve maternal and infant outcomes.^17^

At present, the common methods for labor analgesia are divided into pharmacological analgesia and non-pharmacological analgesia.^18^ Daole analgesia meter relieves pain by mainly using low-frequency D-T electric pulse waves to gently stimulate the peripheral nerves of the body, activate biochemical reactions, promote endogenous opioid peptides during labor, and block the transmission of uterine pain signals to the central brain. Additionally, as a non-invasive technology, it has no adverse effects on the consciousness and uterine smooth muscle of parturients during its use, with a high safety coefficient.

### Limitations

However, this study was conducted only in one hospital, and the evaluation is not comprehensive enough. Therefore, a larger multi-center prospective sample study is needed to avoid bias and these limitations, and more samples should be included and follow-up time should be increased in future studies to further validate the findings of this study.

## CONCLUSIONS

The Daole analgesia meter combined with Daole accompaniment can effectively reduce the labor pain score of High-risk factors Parturients, improve their anxiety, shorten labor duration, and enhance delivery quality. Therefore, it is worth promoting and applying in clinical practice.

### Authors’ Contributions:

**HH** and **BS:** Carried out the studies, participated in collecting data, and drafted the manuscript, and are responsible and accountable for the accuracy and integrity of the work.

**XZ** and **JZ:** Study design, concept, statistical analysis and participated in its design. Critical Review

**TZ: A**cquisition, analysis, or interpretation of data and draft the manuscript.

All authors read and approved the final manuscript.
